# Effect of Alligator Weed (*Alternanthera philoxeroides*) Supplementation on Production Performance, Immune Response and Antioxidant Function of Improved Rural Chicken

**DOI:** 10.3390/ani15050742

**Published:** 2025-03-05

**Authors:** Kekungu-u Puro, Sayed Nabil Abedin, Zakir Hussain, Jaredth B. M. Wankhar, Sunil Doley, Chubasenla Aochen, Burhan Uddin Choudhury, Mahak Singh, Rahul Katiyar, Sourabh Deori

**Affiliations:** 1 Division of Animal and Fisheries Science, Indian Council of Agricultural Research (ICAR) Research Complex for North Eastern Hill Region, Meghalaya 793103, India; akulepuro@rediffmail.com (K.-u.P.); sayedna14@gmail.com (S.N.A.); zakirh8131@gmail.com (Z.H.); jaredthbmwankhar05@gmail.com (J.B.M.W.); sunil.doley@icar.gov.in (S.D.); rahul.katiyarvet@gmail.com (R.K.); 2 Division of Plant Biochemistry, Indian Council of Agricultural Research (ICAR) Research Complex for North Eastern Hill Region, Meghalaya 793103, India; chubasenla.aochen@icar.gov.in; 3 Division of Soil Science, Indian Council of Agricultural Research (ICAR) Research Complex for North Eastern Hill Region, Meghalaya 793103, India; buhan.choudhury@icar.gov.in; 4 Indian Council of Agricultural Research (ICAR) Research Complex for North Eastern Hill Region, Nagaland Centre, Medziphema 797106, India; mahaksinghivri@gmail.com

**Keywords:** alligator weed (AW), immune status, production performance, antioxidant levels, rural poultry

## Abstract

Alligator weed (AW; *Alternanthera philoxeroides*) can have severe ecological and economic consequences by impacting water quality, flow and the growth of native flora and fauna. Plants, both edible and inedible, contain phenolics, compounds that can serve as antioxidants. Using this background information, the study aimed to explore the possible antioxidative nature of AW by incorporating it as a supplement on production performance, gene expression, and antioxidant levels during the summer and winter seasons in improved-variety rural chicks. A total of 400 improved-variety Vanaraja chicks (35 days old), were used in each of the two seasons (summer and winter), making a combined total of 800 birds (*n* = 400 for each summer and winter season). The chicks were subjected to four experimental dietary treatments over a 35-day period during both seasons. The experimental diet consisted of the following: a control diet without any supplements (C); basal diet + 1% AW (T_1_); basal diet + 2% AW(T_2_); and basal diet + 4% AW(T_3_). The production performance, cytokine gene expression (*IFN-γ*, *IL-1β*, *IL-6*, *IL-12* and *iNOS*) and serum antioxidants, viz. catalase (CAT) and superoxide dismutase (SOD), were evaluated. The results indicated that body weight, average body weight gain and weekly feed intake in the T_1_ group was significantly (*p* < 0.05) higher as compared to the other groups. The FCR in group T_1_ was significantly (*p* < 0.05) lower during winter than in summer. A significant (*p* < 0.001) upregulation in the expression of *IL-6*, *IL-1β* and *IL-12* in T_1_ as compared to the other groups was reported. *IFN-γ*, *IL-1β*, *IL-6* and *iNOS* were significantly (*p* < 0.001) upregulated in winter. SOD and CAT activity was significantly (*p* < 0.001) higher in T_1_ compared to C, and both were significantly (*p* < 0.05) higher during winter than in summer. The results suggested that AW has the potential to mitigate the consequences of cold stress on growth, immune response, and antioxidant function during winter. We propose adding 1% AW, which can possibly function as an antioxidant, to the diet of chicks to enhance their production performance and immunity levels.

## Simple Summary

This study addresses the dual challenges of cold stress in rural poultry and the ecological threat posed by alligator weed (AW; *Alternanthera philoxeroides*), an invasive plant. This research aimed to evaluate the effects of incorporating AW as a dietary supplement on production performance, immune responses, and antioxidant levels in Vanaraja chicks during the summer and winter season. The results showed that chickens fed a diet with 1% AW had a noticeably better body weight, weight gain and feed intake, along with a more efficient feed use, especially during winter. These chickens also demonstrated stronger immune responses and better protection against stress, as indicated by higher levels of beneficial immune signals and natural defence enzymes in their bodies. These findings suggest that 1% AW supplementation mitigates the effects of cold stress, enhances productivity and boosts immunity in poultry. By repurposing an invasive weed as a sustainable and cost-effective feed additive, this study offers a solution to improve rural poultry farming while addressing environmental challenges, contributing to food security and ecological sustainability.

## 1. Introduction

Rural poultry farming is a significant source of food and nutritional security in developing countries. Rural poultry provides quality animal protein in the form of eggs and meat to disadvantageous communities. A low initial investment and regular income makes it a lucrative option for poverty alleviation programmes. It also empowers the women in those regions, as rural poultry is mostly looked after and managed by womenfolk. Moreover, it performs several ecosystem services like pest control, manuring, etc. However, rural poultry birds are less productive in terms of egg production and growth rate as compared to commercial birds. Therefore, in recent times there has been significant research into improved rural poultry birds or low-input technology (LIT) birds with enhanced production performances. Nevertheless, the increased feed costs and high mortality in harsh climates put these birds in a disadvantageous condition as compared to local birds. Recently interest in poultry feed supplements has increased, particularly in commercial settings, to improve the feed efficiency, health status and body condition of birds, as well as their productivity. For rural poultry, the choice of feed supplement depends upon economic viability and easy access, as feed is the critical input. The feed supplements used in commercial poultry farming may not be economically feasible in resource-poor settings. Therefore, there is a need to utilise locally available feed supplements to augment health and productivity.

Alligator weed (AW) is one such plant, available everywhere as an invasive weed with potential as livestock feed. A wide adaptability, an ability to grow under stressful conditions and a positive response to global warming are key contributors to the thriving invasion of this weed species [1]. Additionally, it has the ability to thrive in both wetland and damp terrestrial conditions [2]. Previous studies have documented it as a leafy vegetable or substitute for green fodder for dairy animals during different seasons [3,4,5]. The crude protein (CP) levels of AW fall within a satisfactory range [6], and it possesses an outstanding nutritional quality, with significant levels of macronutrients, micronutrients, fat and protein content, even surpassing that of certain commonly used fodders like maize, sorghum and alfalfa [7,8].

Phenolic compounds in plants, edible or not, have diverse effects, including acting as antioxidants, protecting cells from damage. The primary reason behind their antioxidant activity is their ability to function as reducing agents [9]. In general, plants contain phyto-active compounds with anti-inflammatory, immunomodulatory and antioxidant properties. These antioxidant and anti-inflammatory activities are mediated either by directly scavenging radicals or indirectly stimulating antioxidant molecules and anti-inflammatory enzymes or regulating transcription factors to provide a defence against oxidative stress (OS) and mitigate inflammation [10]. In this context, regarding nutritive components, AW is abundant in amino acids and possesses a significant total nitrogen content [11]. It also contains glycosides, tannins, flavonoids and saponins [12] and possesses medicinally valuable water-extractable phytochemicals [13]. The drawbacks associated with the application of herbicides/weedicides to control the weed, such as resistance development, food product residues, harmful effects on grazing and negative impacts on aquatic flora, necessitate the exploration of alternative methods to tackle the problem of aquatic weed infestation [14].

Previous studies on poultry feed supplements have primarily focused on commercial additives, often neglecting their economic feasibility for rural settings. Research on AW has been limited to its use as livestock feed, with no studies exploring its potential as a supplement for poultry, especially improved rural poultry breeds. Additionally, the role of AW in enhancing the immune response and antioxidant status and mitigating seasonal stress remains unexamined. Therefore, we investigated the effect of AW supplementation on the production performance, immune response and antioxidant function of improved rural chickens.

## 2. Materials and Methods

### 2.1. Reagents

Unless indicated otherwise, all chemicals were procured from Sigma-Aldrich(St. Louis, MO, USA).

### 2.2. Ethical Approval

The Institutional Animal Ethics Committee (IAEC) of the Indian Council of Agricultural Research (ICAR) Research Complex for North Eastern Hill Region (NEHR), Umiam, Meghalaya, granted approval for the current investigation.

### 2.3. Experimental Location and Climate Condition

**Figure 1:**
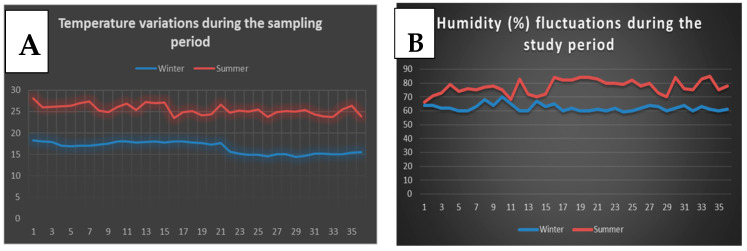
Climatic variables during the study period (35 days). (**A**) Temperature (°C) fluctuations during the sampling period (monthly average—summer: 25.47 ± 1.17 °C; winter: 16.57 ± 1.37 °C). (**B**) Humidity (%) fluctuations during the sampling period (monthly average—summer: 77.38 ± 5.12%; winter: 62.28 ± 2.56%).

The study was conducted at the poultry research farm of the ICAR Research Complex for NEHR, Umiam, Meghalaya, India. The study site is located between 250 40′ N latitude and 910 55′ E longitude (997 m above mean sea level). The yearly temperature ranges from 7.2 to 18.9 °C for the minimum and from 21.3 to 29.6 °C for the highest levels, respectively. The daily temperature (°C) and humidity (%) fluctuations during the study period, viz. summer (June–July) and winter (December–January) months, were recorded in situ and further crosschecked with the State Meteorological Department, Meghalaya, India (Figure 1).

### 2.4. Processing of Alligator Weed and Proximate Analysis

The fresh AW was collected from the vicinity of the institute’s poultry farmhouse. The selection criteria mandated that only green leaves in good condition were selected, while any that were discoloured or dried were removed. The fresh AW was washed, chopped and homogenised with 80% methanol, then filtered to obtain the fresh extract. For the dry extract, the air-dried plant material was powdered, macerated in 80% methanol for 48 h, filtered and concentrated using a rotary evaporator. A proximate analysis of the nutrient composition in the AW and supplemented ration were conducted in triplicate as per AOAC [15]. Proximate components, such as moisture content, dry matter (DM), total ash (TA), crude fibre (CF), crude protein (CP) and ether extract (EE), were evaluated and expressed on a dry weight basis, and nitrogen-free extract (NFE) was calculated as % NFE = 100% − (% EE + % CP + % Ash + % CF).

### 2.5. Experimental Birds and Study Design

The experiment involved 35-day-old chicks, which were fed specially prepared feed mixtures for 35 days. The research used a total of 800 Vanaraja chicks, 400 in the summer season and 400 in the winter season, vaccinated against Marek’s disease, Newcastle disease and infectious bronchitis. The chicks were procured from the ICAR Directorate of Poultry Research (DPR), Hyderabad, India. The chicks were subjected to four experimental dietary treatments during the 35-day trial period during both seasons. The birds were housed at the Experimental Poultry Shed, ICAR RC for the NEH Region, under standard housing system and managemental practices. Each treatment had 5 (five) replicates of 20 birds each. The experimental diet consisted of the following: control diet without any supplements (C); 1% AW (T_1_); 2% AW (T_2_); and 4% AW (T_3_). The basal diet included maize, rice polish (RP), soya and groundnut cake (GNC) and was formulated as per NRC [16] with vitamin and mineral additives (Table 1). All chemicals use in the study were procured from Sigma-Aldrich, unless stated.

### 2.6. Phenolic and Flavonoid Estimation

The phenolic content of the AW was determined using Folin–Ciocalteu technique with minor modifications [17]. In brief, AW extract (1 mL) was placed into a test tube, Folin–Ciocalteu reagent (0.4 mL) was added, and it was set aside for 5–8 min. Then 4 mL of 7% Na_2_CO_3_ was added and mixed well. Next, 10 mL of distilled water (DW) was added, and the mixture was kept for 2 h at 37 °C. Absorbance was determined at 750 nm using UV–Vis spectrophotometry. The results were expressed as gallic acid equivalent (GAE) mgg^−1^ of extract, and a standard curve was plotted using gallic acid (1 mg/mL) at different concentrations (1, 0.50, 0.25, 0.10, 0.05, 0.02, 0.01 and 0 mg/mL).

A colorimetric analysis was used to assess the total flavonoid content [18]. In summary, 1 mL of the sample was added to 3 mL of methanol, followed by the addition of 0.2 mL of 1 M potassium acetate and 0.2 mL of 10% AlCl₃, and then diluted with 10 mL of distilled water. The mixture was set aside for 30 min at 37 °C, and the absorbance was determined at 432 nm. To create a standard curve, quercetin was used at various concentrations (ranging from 0 to 1 mg/mL). The results were presented in terms of quercetin equivalent (QE) mg/g of total extract.

### 2.7. Production Performances

The average daily weight gain (g), average daily feed intake (g) and weekly feed intake (g/wk/bird) were recorded. The feed conversion ratio (FCR) was recorded at weekly intervals for all the groups.

### 2.8. Blood Collection and Isolation of Peripheral Blood Mononuclear Cells (PBMCs)

The blood was drawn aseptically from the jugular vein. For the mononuclear blood cells, the blood was collected in a syringe containing anti-coagulant (heparin @ 40–60 IU/mL). The blood was then diluted with PBS at a 1:1 ratio, and then placed in a 15 mL centrifuge tube that contained an equal volume of histopaque-1077. The tube was then centrifuged (REMI R-8C Centrifuge) for 45 min at 20 °C at 1800 rpm. Mononuclear cells were collected from the gradient’s interface and washed with PBS twice for 10 min each. For serum collection, the blood was drawn into a sterile vial without anti-coagulant and kept at room temperature for some time till the serum oozed out. The serum fraction was separated out by centrifugation at 5000 rpm for 10 min and the supernatant collected.

### 2.9. Estimation of Nitric Oxide (NO) Production—Measure of Oxidative Defence

The production of NO by the PBMCs was measured in an in vitro culture. Isolated mononuclear cells were cultured in RPMI medium (1 × 10^6^ cells/mL concentration), with 5% CO_2_ at 37 °C overnight. The chicken PBMC cultures were treated with either fresh or dried AW extract in triplicate at concentrations of 1, 2 or 4 mg/mL and incubated with a 5% CO_2_ concentration at 37 °C. The cells were then observed at various time points (2, 4, 6 and 24 h), and the supernatant was collected to estimate the production of NO by Griess assay [19]. The cells collected were also used to measure the fold change expression of the *iNOS* gene following the standard qPCR method.

### 2.10. Quantitative Reverse Transcriptase–Polymerase Chain Reaction (qRT-PCR)

The isolated PBMCs were collected, and mRNA was extracted by TRIzol [20]. The concentration and purity of RNA was determined using a NanoDrop 2000 (Thermo Scientific, UK). The RNA was then normalised, and 1 µg of the extracted RNA was reverse-transcribed using the RevertAid First Strand cDNA synthesis kit (Catalogue No. K1621, Thermo Scientific). Following cDNA synthesis, the resulting product was utilised in the qPCR or preserved at −20 °C for later use. A list of primers used in the study has been presented in Table 2. The cytokine (*IL-1β*, *IFN-γ*, *IL-6* and *IL-12*) and *iNOS* expression was checked by qRT-PCR with *β-Actin* as an internal control. The amplified products were detected using the 7500 Fast Real Time PCR system (Applied Biosystems). The amplification efficiency of each target gene, including *β-Actin*, was confirmed to be approximately 100% during the exponential phase of the reaction, where the cycle threshold (Ct) is calculated. The normalised Ct values of the relevant genes were calculated using the average Ct values of the reference control gene (ΔCt), and the relative expression of each representative was determined as 2^−ΔΔCt^ [21].

### 2.11. Estimation of Antioxidant Status

#### 2.11.1. Superoxide Dismutase (SOD)

The SOD activity in serum samples of chickens fed with AW was assessed by the colorimetric method using a commercially available SOD ELISA Kit (Invitrogen, Catalogue No. EIASODC). The serum samples from both the control and treatment groups (C, T1, T2 and T3) were diluted 1:5 in 1X assay buffer. The standard (4 U/mL) provided in the kit was resuspended, and serial dilutions (2 U/mL, 1 U/mL, 0.5 U/mL, 0.25 U/mL, 0.125 U/mL, 0.0625 U/mL and 0 U/mL) were prepared in 75 µL of 1X assay buffer. An amount of 10 µL of each diluted sample and standard was transferred into a 96-well plate provided in the kit. After that, 50 µL of 1X Substrate and 25 µL of 1X Xanthine Oxidase was added to each well as per the manufacturer’s instructions. The plate was incubated for 20 min at room temperature, and the absorbance was read at 450 nm.

#### 2.11.2. Catalase (CAT)

A commercial kit for the colorimetric CAT assay (Invitrogen, Catalogue No. EIACATC) was used to measure the catalase activity in serum samples of chickens fed with AW. Serum samples of both the control and test groups (C, T1, T2 and T3) were diluted in a 1:5 ratio with 1X assay buffer. The standard (100 U/mL, provided in the assay kit) was diluted to prepare serial dilutions (5 U/mL, 2.5 U/mL, 1.25 U/mL, 0.625 U/mL, 0.313 U/mL, 0.156 and 0 U/mL) of 100 µL each in 1X assay buffer. A 96-well plate (provided in the kit) was used to transfer 25 µL each of the diluted samples and standards. Then, 25 µL of hydrogen peroxide was added into each well, and the plate was incubated for 30 min at room temperature. Substrate (25 µL) was added in each well, followed by 25 µL of 1X HRP solution in all wells, and the plate was further incubated for 15 min at room temperature. The absorbance was measured at 560 nm.

### 2.12. Liver Function Tests

The serum activities of hepatic aspartate aminotransaminase (AST), alkaline phosphatase (ALP), alanine aminotransferase (ALT), albumin and total proteins (TPs) as indicators of liver damage were measured by spectrophotometric analysis using commercial kits purchased from Bioline Diagnostics Llp (New Delhi, India) at 1 week and 1 month intervals of blood collection from the experimental birds fed 1% AW in their rations.

### 2.13. Statistical Analysis

The datasets generated were checked for normality, and a data analysis was carried out using the statistical program SPSS (ver. 23.0, SPSS Inc., Chicago, IL, USA), using a one-way ANOVA followed by Duncan’s multiple comparison tests within the different treatments in the same season to check significant differences. A two-way ANOVA was performed to evaluate the effects of treatment and season, as well as their interaction, on the measured parameters. The interaction effect between treatment and season was tested and found to be non-significant (*p* > 0.05), which justified the interpretation of the main effects of treatment and season independently. Results were considered statistically significant at *p* < 0.05.

## 3. Results

### 3.1. Proximate Component Analysis of AW and the Experimental Ration

The proximate analysis of the AW sample revealed a notable nutrient composition (Table 3). The TA content was 18.48 ± 0.40%. The DM content was 85.47 ± 0.40%, with an OM content of 81.53 ± 0.26%. CP was measured at 14.16 ± 1.42%, while the crude fat content was 4.83 ± 0.51%. The CF content was relatively high at 13.92 ± 1.34%, and the NFE was recorded at 48.74 ± 2.55%, reflecting the carbohydrate fraction of the sample.

The proximate components of the experimental ration in comparison to the control ration have been presented in Table 4. The CP and crude fat content remained statistically (*p* > 0.05) similar among all groups, indicating that AW supplementation did not affect these parameters. However, CF showed a significant (*p* < 0.05) increasing trend with a higher AW inclusion, with T_3_ (6.50 ± 0.15%) having the highest CF, significantly different from the control (4.50 ± 0.40%) and T_1_ (4.00 ± 0.30%). NFE levels did not show significant (*p* > 0.05) variation among the groups, suggesting a stable carbohydrate content across diets. These results indicate that AW supplementation primarily influenced moisture, DM, TA and CF levels without significantly altering CP, crude fat, OM or NFE content.

### 3.2. Phenolic and Flavonoid Concentration

The phytochemical component (phenolic and flavonoid) of the AW has been presented in Table 5. The total phenolic content as per our analysis was 2.23 ± 0.32 mg GAE/g, and the flavonoid content was 1.44 ± 0.19 (SE) mg QE/g of fresh weed.

### 3.3. Production Performances

**Figure 2:**
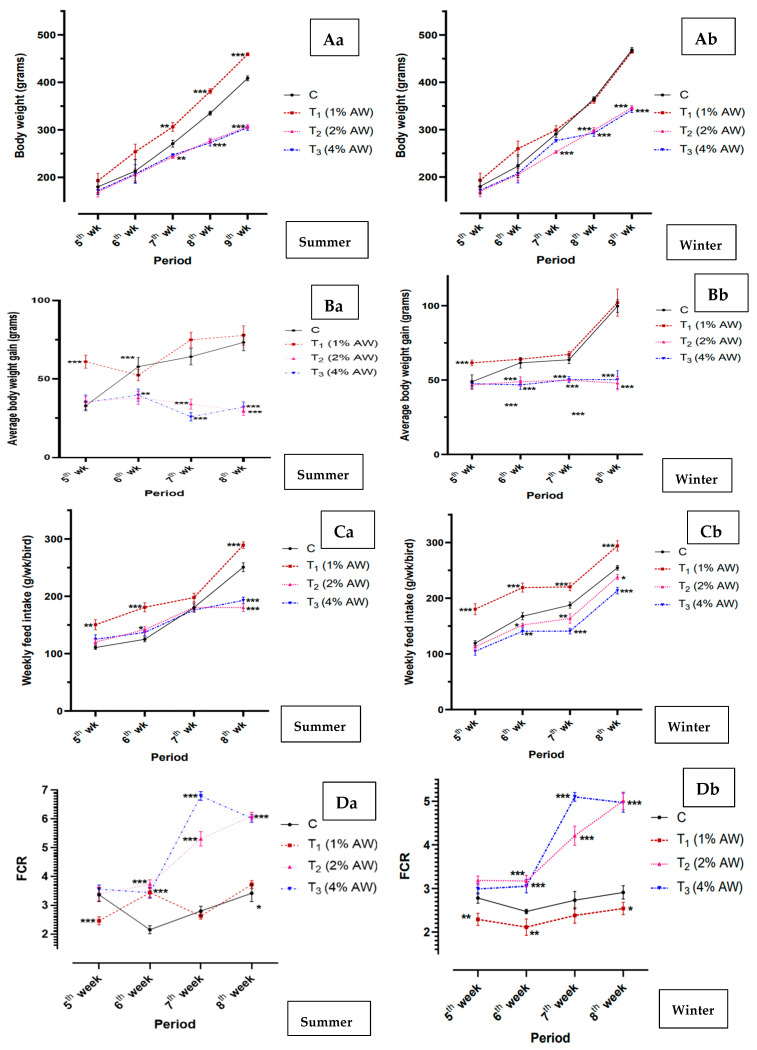
Production performances of chicks (day 35 to 77) fed with experimental ration during summer and winter. (**Aa**,**Ab**) Body weight (g). (**Ba**,**Bb**) Average body weight gain (g). (**Ca**,**Cb**) Weekly feed intake per bird (g/wk). (**Da**,**Db**) Feed conversion ratio (FCR). * *p* < 0.05; ** *p* < 0.01; *** *p* < 0.001 [The *p* values were arrived at par in comparison to control group]. Abbreviations: AW—Alligator weed; FCR—Feed conversion ratio.

The production performance, viz. for body weight (Figure 2Aa—summer: Figure 2Ab—winter), average body weight gain (Figure 2Ba—summer: Figure 2Bb—winter), weekly feed intake (Figure 2Ca—summer: Figure 2Cb—winter) and FCR (Figure 2Da—summer: Figure 2Db—winter) of the experimental birds fed AW at different levels (1%, 2% and 4%) has been compiled in Figure 2. As is evident from our findings, the body weight (g), average body weight gain (g) and weekly feed intake (g/wk/bird) was significantly (*p* < 0.05) higher in T_1_. The weekly feed intake (g/wk/bird) was significantly (*p* < 0.001) higher during winter as compared to summer in T_1_. The FCR was significantly (*p* < 0.001) lower in T_1_, especially during winter.

### 3.4. Effect of AW Extract on NO Production and iNOS Gene Expression

**Figure 3:**
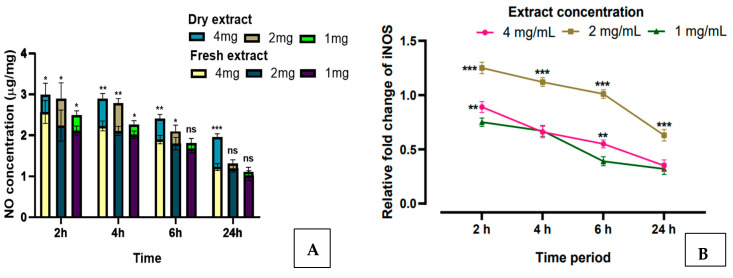
In vitro effects of AW extract (4, 2 and 1 mg) on NO production and *iNOS* gene expression at different time periods (2, 4, 6 and 24 h). (**A**) Effect of AW extract (dry vs. fresh) on NO concentration in vitro. (**B**) In vitro effect of AW extract concentration (4, 2 and 1 mg) on relative fold change of *iNOS* gene at different time periods. Abbreviations: AW—Alligator weed; NO—Nitric oxide; *iNOS*—Inducible nitric oxide synthase. * *p* < 0.05; ** *p* < 0.01; *** *p* < 0.001; ns—Non-significant.

Figure 3A,B show the effect of AW extract on in vitro NO generation (µg/mL) and *iNOS* gene expression. As is evident from our findings, the production of NO is extract concentration-dependent, and NO production decreased with a decreasing extract concentration. The concentration of NO was significantly (*p* < 0.001) higher with the dry extract as against the fresh extract, which might be due to a greater concentration of active components in the dry extract, while the fresh extract had a higher moisture content (Figure 3A). The expression of the *iNOS* gene, which is responsible for synthesising NO in the body, was significantly (*p* < 0.001) upregulated in PBMC culture treated with the dry AW @ 2 mg/mL concentration in 2 h of incubation time in comparison to other extract concentrations (Figure 3B).

### 3.5. Relative Expression Profile of Innate Immune Marker Genes

**Figure 4:**
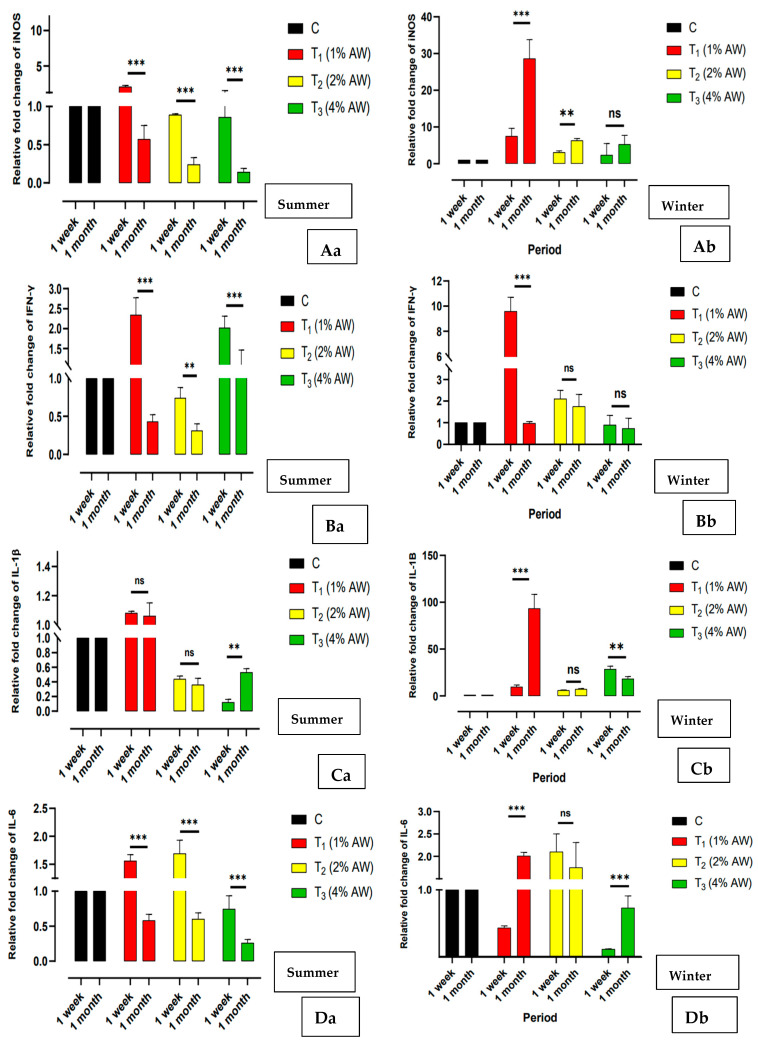

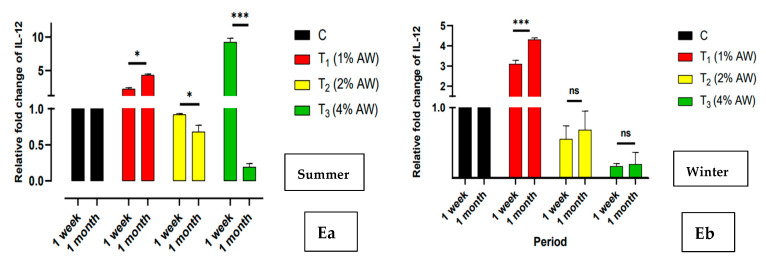
Graphical representation of the fold change expression of innate immune marker genes in the different treatments (day 35 to 77 of feeding AW) and in the different study seasons. (**Aa**,**Ab**) Relative fold change of *iNOS*. (**Ba**,**Bb**) Relative fold change of *IFN-γ.* (**Ca**,**Cb**) Relative fold change of *IL-1β*. (**Da**,**Db**) Relative fold change of *IL-6*. (**Ea**,**Eb**) Relative fold change of *IL-12*. *** *p* < 0.001; ** *p* < 0.01; * *p* < 0.05; ns—Non-significant. Abbreviations: AW—Alligator weed.

The relative fold change expression of *iNOS* (Figure 4Aa—summer: Figure 4Ab winter), *IFN-γ* (Figure 4Ba—summer: Figure 4Bb winter) and innate immune marker genes, viz. *IL-1β* (Figure 4Ca—summer: Figure 4Cb winter), *IL-6* (Figure 4Da—summer: Figure 4Db winter) and *IL-12* (Figure 4Ea—summer: Figure 4Eb winter), in the PBMCs at two different time periods during both the study seasons has been presented. The current results revealed a significant (*p* < 0.001) upregulation in the expression of *IL-1β*, *IL-6* and *IL-12* in group T_1_ in comparison to the control. Furthermore, in a comparison between seasons, the expression of *iNOS, IL-6* and *IL-1β* was significantly (*p* < 0.001) upregulated by many folds in winter in comparison to summer. A similar trend was reported in the expression profile of the *IFN-γ* gene.

### 3.6. Antioxidant Activity

**Figure 5:**
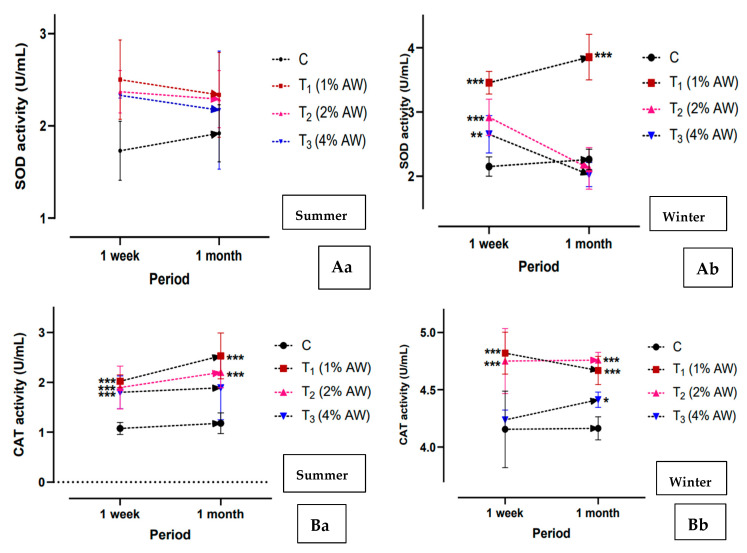
Effects of feeding AW on the production of antioxidant levels in serum. (**Aa**,**Ab**) SOD activity (U/mL), (**Ba**,**Bb**) CAT activity (U/mL). *** *p* < 0.001; ** *p* < 0.01; * *p* < 0.05. Abbreviations: SOD—Superoxide dismutase; CAT—Catalase.

The antioxidant activity, viz. SOD (Figure 5Aa—summer: Figure 5Ab—winter) and CAT (Figure 5Ba—summer: Figure 5Bb—winter) activity, in the different treatments has been presented. As per our findings, CAT activity significantly (*p* < 0.001) increased during winter months in comparison to summer, and significantly (*p* < 0.001) higher activity was recorded in group T_1_ as against C. SOD activity was significantly (*p* < 0.001) higher in T_1_ in comparison to C during winter, but no significant (*p* > 0.05) difference was recorded during summer.

### 3.7. Liver Function Tests

**Figure 6:**
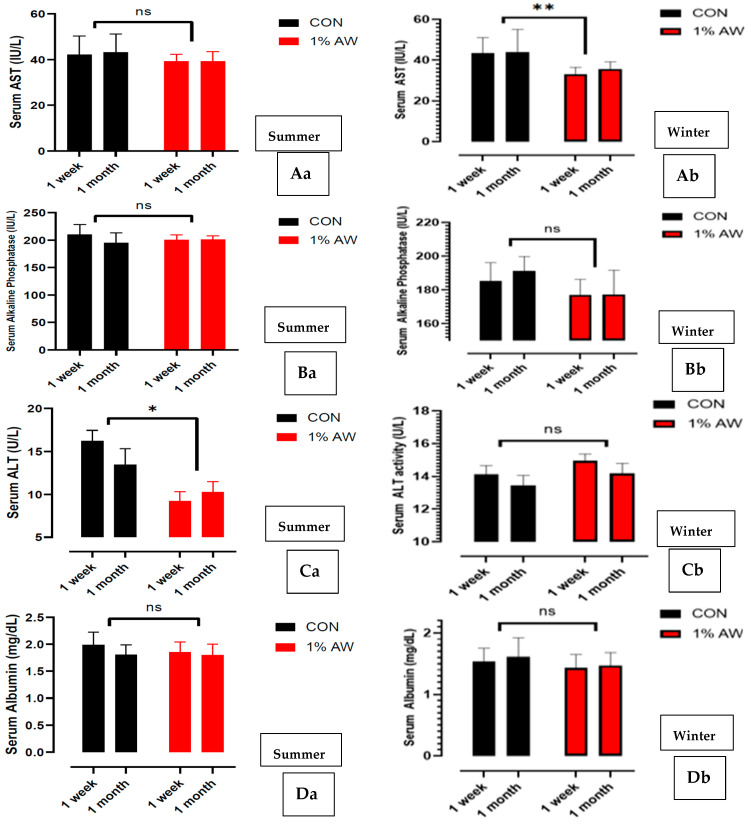

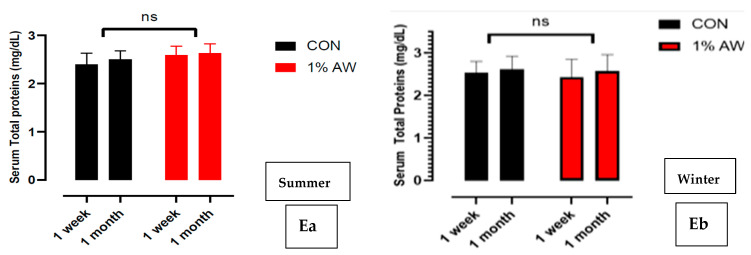
Hepatic liver enzyme activity in serum samples of chicks fed with 1% AW supplementation during summer and winter. (**Aa**,**Ab**) Serum AST activity (IU/L). (**Ba**,**Bb**) Serum alkaline phosphatase levels (IU/L). (**Ca**,**Cb**) Serum ALT activity (IU/L). (**Da**,**Db**) Serum albumin levels (mg/dL). (**Ea**,**Eb**) Serum total proteins (mg/dL). * *p* < 0.05; ** *p* < 0.01; ns—Non-significant (*p* > 0.05) [The *p* values were arrived at par in comparison to control group]. Abbreviations: AW—Alligator weed; AST—Aspartate aminotransaminase, ALT—Alanine aminotransferase.

The antioxidant activity, viz. AST (Figure 6Aa—summer: Figure 6Ab—winter), ALP (Figure 6Ba—summer: Figure 6Bb—winter), ALT (Figure 6Ca—summer: Figure 6Cb—winter), albumin (Figure 6Da—summer: Figure 6Db—winter) and TP (Figure 6Ea—summer: Figure 6Eb—winter) levels in the best treatment (1% AW), has been depicted. The current results depicted significantly (*p* < 0.01) lower serum AST levels in the 1% AW-supplemented group in the winter season in comparison to CON. In contrast, serum ALT levels were significantly (*p* < 0.05) lower in the treatment group (1% AW) in summer in comparison to winter. No significant (*p* > 0.05) changes were reported in the levels of serum ALP, albumin and TP in both the study seasons in the treatment T_1_ in comparison to the control group.

## 4. Discussion

The invasive presence of AW has been observed in numerous aquatic bodies in India, it having spread to multiple states. The displacement of native vegetation by AW, which is typical of many invasive aquatic plants, makes it a nuisance for the farming community. Hence, an effective way to utilise these weed plants is by utilising them as livestock and poultry feed, since transforming agricultural waste into useful products can be an eco-friendly, cost-effective and sustainable method of waste management. In this regard, we investigated the effects of the supplemental feeding of AW on the production performances, immune status and antioxidant profile in growing chicks during existing summer and winter seasons under standard housing and management conditions. To the best of our knowledge, this current study is the first to validate the antioxidant and stress-alleviating properties of wild AW fed to growing chicks under existing managemental conditions, proving it valuable as a poultry feed supplement and as an effective alternative for sustainable waste management in changing climatic conditions.

A proximate analysis of feed is a vital criterion for measuring food nutritional values and quality [22]. A few workers reported that the moisture content of AW ranges from 85% in terrestrial weed [4] to 90.10% in aquatic weed [23]. This discrepancy in moisture content and DM could have resulted from the harvesting stages of the weeds, as the moisture content is known to decline with maturity and can differ across habitats. The CP content (14.16%) was a little lower as compared to the 16.30% in terrestrial weed reported by Kumar and Vishwakarma [4]. However, the NFE (48.74%) was higher as compared to the 36.60% found in aquatic AW [23].

Polyphenols, flavonoids and tannins, among other secondary metabolites or phytochemicals, are present in almost all plants, albeit in varying quantities. Aside from contributing to a plant’s colour, flavour and functionality, these compounds possess strong antioxidant properties that can alleviate OS by capturing free radicals through the donation of electrons. These compounds can lower the levels of various free radicals, including hydrogen peroxide, superoxide radicals and hydroxyl radicals [24,25,26]. Previous studies have validated a link between total phenol content and antioxidant activity [27,28]. The current results on the phenolic content of AW are significantly lower than the values reported by Kumar and Vishwakarma [4] and Tukun [29]. Such a discrepancy in the phenolic content of AW extract may stem from the solvents chosen for the extraction, as solvents with a higher polarity are inclined to yield greater quantities of polyphenolic compounds [30]. Furthermore, it has been reported that variations in the levels of polyphenolic compounds observed in various studies could be linked to differences in the geographic location and time of harvest, as environmental variables may impact the flavonoid content [31]. Our reported values for flavonoid content are lower than the values reported by Khandker [32] but are within the range of 0.0187 to 3.2 mg/g as reported in previous studies [29,33]. The flavonoid content of several plant species is significantly impacted by genetic diversity, environmental, biological and yearly seasonal variations, according to the existing literature [34].

The evaluation of poultry growth status under diverse conditions necessitates the use of growth performance as a vital indicator [35]. The findings of this study indicated a markedly elevated weekly feed intake in group T_1_, especially during the winter season, in contrast to the other groups. These results suggest that a diet containing 1% AW may impact signal receptors involved in appetite control and may prove effective in mitigating stress-induced anorexia during extreme cold stress. Conversely, the addition of 2% and 4% AW resulted in the reduced body weight of the chicks, accompanied by a lower feed intake compared to the control group. Overall, our study’s results indicate that 1% AW can be satisfactorily incorporated into the diet of poultry birds raised under intensive deep litter housing, given the satisfactory CP content and its effects on production performance. Nevertheless, it is imperative to conduct further research on its potentiality when used as a replacement in part or whole against major ingredients and on its antinutritional elements, which may lead to reduced palatability, mineral bioavailability and protein digestibility and, ultimately, limit the biological value and acceptance of AW as a regular food source for poultry feeding.

The role of NO as an endothelial vasodilator during thermal stress, which is characterised by abrupt temperature variations, is well established [36,37]. It enhances autonomic heat dissipation by stimulating peripheral blood flow. NO primarily exerts its biological effects through the activities of NOS, which includes the inducible NOS (*iNOS*) isoforms, neuronal NOS (*nNOS*) and endothelial NOS (*eNOS*) [38]. Additionally, when exposed to heat, the level of *iNOS* expression was found to be enhanced [39]. The outcomes of our research reveal that the expression of the *iNOS* gene, responsible for generating NO in the body, was significantly upregulated in PBMC culture treated with dry AW at a concentration of 2 mg/mL. NO has several advantageous effects, but it also contributes to oxidative damage, which is primarily due to its interaction with superoxide ions, resulting in the formation of a peroxynitrite anion (a potent oxidant) that breaks down into NO_2_ and OH [40]. Our results showed that both fresh and dry AW extract possess robust NO-scavenging abilities, effectively transforming NO into nitrites. Our results are in line with the reports of Teshfam [41] and Zhang [42], wherein *iNOS* expression was upregulated in the duodenum and lungs of birds during chronic cold stress. Even though we did not measure lipid peroxide levels in our investigation, it is critical to note that cold stress can impair the antioxidant defence system in living organisms, resulting in lipid peroxidation products, ultimately causing cell necrosis, thereby releasing inflammatory mediators. These mediators can then stimulate *iNOS* expression, leading to an excessive NO release. As demonstrated in the previous sections, the NO-scavenging abilities identified in our study could also be ascribed to secondary metabolites in these plant extracts, including phenols and flavonoids, which is consistent with Molyneux’s [43] conclusions.

Concerning the production of cytokines, which are used to gauge the immune system status, our research revealed a substantial rise in *IFN-γ* levels in the experimental chicks that consumed a 1% AW diet during winter as opposed to summer. This was unlike the control and other treatment groups. Pro-inflammatory cytokines, specifically *IFN-γ*, play an important role in tissue immune response and the onset of inflammation. To the best of our understanding, this is the first investigation that studies the presence of inflammation-related genes in the blood of chicks that were given different amounts of AW (1%, 2% and 4%). Moreover, our results demonstrated that the treatment group fed with 1% AW had significantly upregulated levels of *IL-6, IL-12* and *IL-1β* during cold stress as compared to the group in the summer. It is conceivable that the decline in ambient temperature during cold stress may have a detrimental impact on chicks, prompting their bodies to generate pro-inflammatory cytokines. Our results are consistent with a few published reports. Brenner [44] found that human plasma *IL-6* levels were elevated with cold exposure preceding exercise. Serum *IL-6* levels in Wistar rats were heightened as a result of exposure to cold stress [45]. Furthermore, it was also reported that *IL-6* expression may relieve the effects of cold stress [46]. In addition, alterations in environmental variables may result in an upsurge in pro-inflammatory cytokines [47]. Flavonoids derived from Chinese herbs can ameliorate immunological stress and enhance immune function in broilers that have been exposed to lipopolysaccharide challenge [48]. It has also been documented in previous studies that plant leaves rich in metabolites such as phenolics, alkaloids, glycosides and flavonoids have immunostimulant and antioxidant potential [49], and a similar bioactive compound of Moringa Oleifera [50,51] and *Pulicaria jaubertii* [52] leaves could enhance the antioxidative status and upregulate immune gene expression in broilers. So, it is possible to assume that the immunostimulant properties of AW can be attributed to its chemical composition.

In the context of intensive chicken farming, birds are subjected to multiple stressors such as imbalanced diets and environmental extremities, which can disturb body homeostasis, causing OS and reduced performance [53]. Antioxidant enzymes, such as GPx, SOD or CAT, as well as other antioxidants, can counteract and guard against OS resulting from reactive oxygen species (ROS) [54]. Recent studies indicate that the defensive outcomes of phytochemicals in poultry are in part due to the activation of Nrf2, which stimulates antioxidant enzyme activity [55]. The current study reported enhanced serum CAT and SOD activity during winter in comparison to summer, and the highest activity was recorded with a 1% AW supplementation. Our investigation is noteworthy in that it is the first to display the antioxidative characteristics of AW, as suggested by the levels of antioxidant enzymes we recorded during the winter period. While no previous investigations have examined the antioxidative potential of AW, it is possible to theorise that elevated levels of serum antioxidants during extreme winter conditions may offer cytoprotective benefits against excessive superoxide generation. There appears to be a link between the heightened activity of antioxidant enzymes and diminished peroxide levels [56]. Lowering MDA levels and boosting CAT and SOD activity, as evidenced by an improved antioxidant status in cold-stressed chicks, is a vital effect of supplementing 1% AW that might help in maintaining the equilibration between oxidants and antioxidants for cellular homeostasis.

AST and, especially, ALT are good predictors of liver injury. Elevated levels of these enzymes in serum are usually caused by hepatocyte necrosis or altered membrane permeability [57]. The liver contains high levels of ALT and AST enzymes. The destruction of the liver leads to the release of these enzymes in the blood stream and subsequently elevates enzyme levels, and increased levels of these enzymes signifies the liver damage condition [58]. The current results depicted lower AST levels in the 1% AW-supplemented group, signifying that the antioxidative nature of AW can ameliorate oxidative damage to hepatic cells. In line with our findings, an in vivo study in mice indicated that antioxidants ameliorated liver damage by reducing the levels of alcohol-metabolising factors and AST, ALT, triglycerides (TGs) and total cholesterol (TC) content, which demonstrated that antioxidants effectively mitigated liver injury in mice [59].

The results of this study indicate that while a 1% AW inclusion demonstrated beneficial effects, higher inclusion levels of 2% and 4% AW were not only ineffective but also exhibited negative impacts. This trend suggests a dose-dependent response to AW supplementation, which warrants further exploration. The adverse effects observed at higher inclusion levels could be attributed to the presence of anti-nutritional factors or bioactive compounds inherent in AW that can interfere with nutrient absorption, enzyme activity and metabolic processes, potentially leading to compromised growth performance and physiological functions, which needs further exploration. Additionally, the increased CF content associated with higher AW inclusion levels might have contributed to reduced nutrient digestibility and feed efficiency. The negative effects observed at the 2% and 4% AW inclusion levels underscore the need for careful dose optimisation. Future studies should focus on elucidating the specific anti-nutritional components responsible for these effects and exploring strategies to mitigate their impact, such as processing methods to reduce harmful compounds or combining AW with other dietary additives to counteract potential negatives.

Our study uniquely evaluates the effect of supplementing AW across summer and winter seasons, addressing oxidative stress and cold stress, which are significant challenges in poultry farming. By focusing on AW’s potential antioxidant properties and its influence on cytokine gene expression, the research provides a novel, sustainable and cost-effective solution for enhancing rural poultry productivity. By repurposing AW, the research not only aims to enhance the health, growth and productivity of rural poultry but also provides an eco-friendly solution to managing invasive weeds, contributing to sustainable agricultural practices and improved livelihoods in resource-poor communities.

## 5. Conclusions

In summary, a dietary 1% AW has the potential to prevent the suppression of growth performance, immune response and antioxidant function, particularly in the face of cold stress, by serving as a potential antioxidant. This has far-reaching implications for potential novel approaches to counteract the negative impact of stress on the performance of grower chicks. If implemented in traditional poultry-feeding systems, these techniques can serve as an environmentally friendly and sustainable option for effective weed management, while also providing valuable fodder/feed supplements for livestock. Nonetheless, before suggesting an appropriate feed formulation with supplementation for poultry feeding that can be endorsed by farmers, it is crucial to clarify the impact of anti-nutritional factors, toxicity or soil quality on its nutrient content and its effectiveness in heat stress conditions, as well as determining the optimal concentration for efficient utilisation.

## Figures and Tables

**Table 1:** Composition and nutrient levels of the basal diet, on an as-fed basis unless stated otherwise (%).

Sl No.	Ingredients	Parts
1.	Maize	56
2.	Rice polish (RP)	10
3.	Soya	16
4.	Ground nut cake (GNC)	15
5.	Mineral mixture (Agrimin ^¥^: 0.5 kg; Gromin-P ^€^: 2 kg)	2.5
6.	Salt	0.5
	Total	100

^¥^—Nutritive value per kg: Vitamin A—7,00,000 IU, Vitamin E—250 mg, Vitamin D3—70,000 IU, Cobalt—150 mg, Iodine—325 mg, Iron—1500 mg, Nicotinamide—1000 mg, Magnesium—6000 mg, Potassium—100 mg, Sodium—5.9 mg, Copper—1200 mg, Manganese—1500 mg, Zinc—9600 mg, Sulphur—0.72%, Phosphorus—12.75%, Calcium—25%. ^€^—Nutritive value per kg: Phosphorus—Min. 9%, Iodine—Min. 0.01%, Moisture Max—3%, Iron Min—2000 ppm, Fluorine Max.—0.05%, Calcium Min.—30%, Acid Insoluble Ash Max—3%, Zinc Min.—0.40%, Selenium Max—3.5 ppm, Manganese Min—0.40%, Copper Min—500 ppm.

**Table 2:** Details of primer sequences used for quantitative real-time PCR analysis.

**Sl. No.**	**Gene**	**Sequence**	**Accession No.**
1	*IL-1β*	F: GGGACTTTGCTGACAGCGACCTG R: GTCGAAGGACTGTGAGCGGGTGT	AJ245728
2	*Β-actin*	F: GCACCACACTTTCTACAATAG R: ACGACCAGAGGCATACAGG	L08165
3	*IL-6*	F: GCTCGCCGGCTTCGA R: GGTAGGTCTGAAAGGCGAACAG	AJ250838
4	*IFN-γ*	F: GCCGCACATCAAACACATATCT R: TGAGACTGGCTCCTTTTCCTT	NM_205149
5	*iNOS*	F: AGGCCAAACATCCTGGAGGTC R: TCATAGAGACGCTGCTGCCAG	U46504
6	*IL-12*	F: TGGTCCACGCTTTGCAGAT R: AAGGTTAAGGCGTGGCTTCTTA	AJ564201

**Table 3:** Proximate analysis of alligator weed (mean ± SE).

TA	DM	OM	CP	EE	CF	NFE
18.48 ± 0.40	85.47 ± 0.40	81.53 ± 0.26	14.16 ± 1.42	4.83 ± 0.51	13.92 ± 1.34	48.74 ± 2.55

Each value represents mean ± SE of three determinants on dry weight (DW) basis. Abbreviations: TA—Total ash; DM—Dry matter; OM—Organic matter; CP—Crude protein; EE—Ether extract; CF—Crude fibre; NFE—Nitrogen-free extract.

**Table 4:** Proximate analysis of experimental ration compared with control ration (mean ± SE).

Particulars (% DM Basis)	C (Control)	T_1_ (1% AW)	T_2_ (2% AW)	T_3_ (4% AW)
Moisture	6.37 ^a^ ± 0.13	10.58 ^b^ ± 0.64	10.09 ^b^ ± 0.50	10.13 ^b^ ± 0.10
DM	93.62 ^b^ ± 0.13	89.67 ^a^ ± 0.89	89.90 ^a^ ± 0.50	89.85 ^a^ ± 0.07
TA	9.20 ^a^ ± 0.80	9.30 ^a^ ± 0.20	8.63 ^a^ ± 0.58	9.37 ^b^ ± 0.95
OM	90.80 ^a^ ± 0.80	90.70 ^a^ ± 0.20	91.37 ^a^ ± 0.58	89.45 ^a^ ± 0.95
CP	20.20 ^a^ ± 1.30	20.91 ^a^ ± 1.08	20.35 ^a^ ± 1.11	19.69 ^a^ ± 1.21
Crude fat	4.55 ^a^ ± 0.55	4.80 ^a^ ± 0.75	4.75 ^a^ ± 0.15	4.63 ^a^ ± 0.40
CF	4.50 ^ab^ ± 0.40	4.00 ^a^ ± 0.30	5.50 ^bc^ ± 0.15	6.50 ^c^ ± 0.15
NFE	61.55 ^a^ ± 2.96	60.99 ^a^ ± 0.79	60.77 ^a^ ± 2.30	59.81 ^a^ ± 1.66

^a,b,c^ Different superscripts differ significantly (*p* < 0.05). Abbreviations: AW—Alligator weed; DM—Dry matter; TA—Total Ash; OM—Organic matter; CP—Crude protein; EE—Ether extract; CF—Crude fibre; NFE—Nitrogen-free extract.

**Table 5:** Biochemical analysis of phytochemical content (phenolic and flavonoid) of AW.

Phenolic content:	2.23 ± 0.32 mg GAE/g fresh weed
Flavonoid content:	1.44 ± 0.19 mg QE/g fresh weed

Abbreviations: GAE—Gallic acid equivalent; QE—Quercetin equivalent.

## Data Availability

The datasets generated during and/or analysed during the current study are available from the corresponding author on reasonable request.
